# Breast cancer statistics for Japan in 2022: annual report of the national clinical database-breast cancer registry—clinical implications including chemosensitivity of breast cancer with low estrogen receptor expression

**DOI:** 10.1007/s12282-025-01671-0

**Published:** 2025-02-06

**Authors:** Masayuki Nagahashi, Hiraku Kumamaru, Naoko Kinukawa, Takayuki Iwamoto, Masahiro Kawashima, Takayuki Kinoshita, Takaaki Konishi, Yasuaki Sagara, Shinsuke Sasada, Shigehira Saji, Naoko Sanuki, Kenta Tanakura, Naoki Niikura, Minoru Miyashita, Masayuki Yoshida, Takanori Ishida, Naruto Taira

**Affiliations:** 1 Department of Surgery, Division of Breast and Endocrine Surgery, School of Medicine, Hyogo Medical University, 1-1 Mukogawa-Cho, Nishinomiya, Hyogo 663-8501 Japan; 2 Department of Healthcare Quality Assessment, The University of Tokyo Graduate School of Medicine, 7-3-1 Hongo, Bunkyo-Ku, Tokyo, 113-8655 Japan; 3 Breast and Thyroid Surgery, Kawasaki Medical School Hospital, 577 Matsushima, Kurashiki City, Okayama 701-0192 Japan; 4 Department of Breast Surgery, Graduate School of Medicine, Kyoto University, 54 Shogoin-Kawahara-Cho, Sakyo-Ku, Kyoto, 606-8507 Japan; 5 Department of Breast Surgery, National Hospital Organization Tokyo Medical Center, 2-5-1, Higashigaoka, Meguro-Ku, Tokyo, 152-8902 Japan; 6 Department of Breast and Endocrine Surgery, Graduate School of Medicine, The University of Tokyo, 7-3-1 Hongo, Bunkyo-Ku, Tokyo, 113-8655 Japan; 7 Department of Breast and Thyroid Surgical Oncology, Hakuaikai Medical Corporation, Sagara Hospital, 3-28 Matsubara, Kagoshima, 892-0833 Japan; 8 Department of Surgical Oncology, Research Institute for Radiation Biology and Medicine, Hiroshima University, 1-2-3 Kasumi, Minami-Ku, Hiroshima, Hiroshima 734-8551 Japan; 9 Department of Medical Oncology, Fukushima Medical University, 1 Hikarigaoka, Fukushima, 960-1295 Japan; 10 Department of Radiology, Keio University School of Medicine, 35 Shinanomachi, Shinjuku-Ku, Tokyo, 160-8582 Japan; 11 Plastic and Reconstructive Surgery, Mitsui Memorial Hospital, 1 Kanda-Izumicho, Chiyoda-Ku, Tokyo, 101-8643 Japan; 12 Department of Breast Oncology, Tokai University School of Medicine, 143 Shimokasuya, Isehara, Kanagawa 259-1193 Japan; 13 Department of Breast and Endocrine Surgical Oncology, Tohoku University School of Medicine, Seiryo-Machi, Aoba-Ku, Sendai, 980-8574 Japan; 14 Department of Diagnostic Pathology, National Cancer Center Hospital, 5-1-1 Tsukiji, Chuo-Ku, Tokyo, 104-0045 Japan

**Keywords:** Japanese Breast Cancer Society, Registry, National clinical database, Annual report, Breast neoplasm

## Abstract

This is an annual report by the Japanese Breast Cancer Society, which provides statistics on the clinical data on breast cancer in Japan, extracted from the National Clinical Database-Breast Cancer Registry (NCD-BCR). This report includes an update of 102,453 breast cancer cases at 1339 institutions registered in the NCD-BCR in 2022. Among the 101,793 female patients, the median age at cancer diagnosis was 62 years (interquartile range, 50–73 years), and 29.4% of the patients were premenopausal. Of these patients, 15,437 (15.2%) and 42,936 (42.2%) were diagnosed with stage 0 and I disease, respectively. Estrogen receptor, progesterone receptor, and human epidermal growth factor receptor 2 (HER2) were positive in 78.7%, 69.4%, and 12.8% of the patients, respectively. Of the 97,154 patients without distant metastasis, 40,521 (41.7%) underwent breast-conserving surgery, and 5780 (5.9%) patients underwent some form of breast reconstruction procedures at the time of mastectomy. A total of 66,894 (68.9%) patients were treated with sentinel lymph node biopsy and 7155 (7.4%) patients were treated with sentinel lymph node biopsy followed by axillary node dissection. In the group of patients treated with breast-conserving surgery (*n* = 40,521), 29,500 (72.8%) received whole-breast irradiation. In the group of patients who underwent mastectomy (*n* = 54,476), 6226 (11.4%) received radiation therapy to the chest wall. Of the 13,950 patients receiving preoperative chemotherapy with or without molecular targeted therapy, 4308 (30.9%) achieved a pathological complete response, with the highest rate of 60.5% in patients with the hormone receptor-negative/HER2-positive subtype.

**Supplementary Information:**

The online version contains supplementary material available at 10.1007/s12282-025-01671-0.

## Preface

The Japanese Breast Cancer Society started the Breast Cancer Registry in 1975. Since 2004, it has been organized as a new web-based system in collaboration with the non-profit organization, Japan Clinical Research Support Unit, and the Public Health Research Foundation (Tokyo, Japan). Subsequently, the management of the breast cancer registry was transferred to the National Clinical Database (NCD) registry platform in 2012. Details of this system have been described previously [1]. Patients diagnosed with new-onset breast cancer at NCD-participating institutions throughout Japan were eligible for the registry, regardless of whether or not they underwent breast surgery. Since the current platform was launched in 2012, the National Clinical Database-Breast Cancer Registry (NCD-BCR), the total number of records has accumulated to 892,021 in the decade from 2012 to 2021 [2–5].

The NCD-BCR is referenced when Japanese physicians obtain or renew their professional certification for themselves or their institution. In addition, a number of studies using the NCD-BCR to address unmet clinical needs have been reported [6–20]. Together, JBCS and the NCD-BCR aim to improve the quality of medical care by sharing data on quality indicators with breast cancer treatment providers.

This report is an NCD-BCR update of 102,453 breast cancer cases registered in 2022 at 1339 institutions. Here, we present the demographic and clinicopathological characteristics of the registered breast cancer patients.

## Selected findings

### Demographics and patient characteristics

**Fig. 1:**
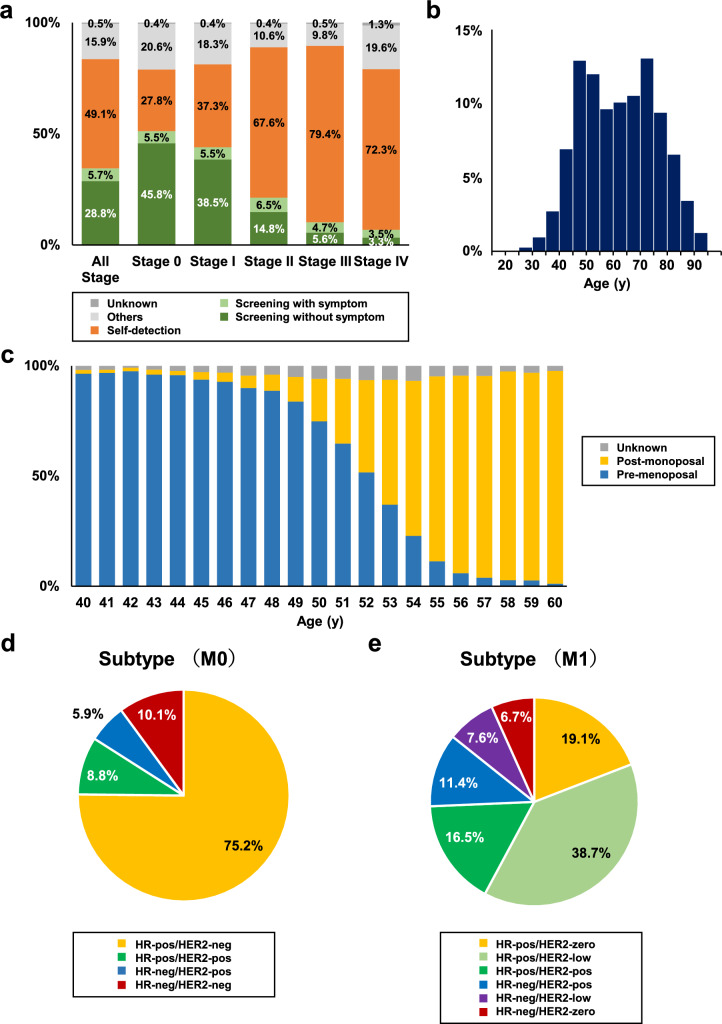
Clinicopathological characteristics of female patients with breast cancer in 2022. **a** Breakdown of opportunities for breast cancer detection is shown by stage. **b** The histogram shows a distribution of the age at cancer diagnosis. **c** Age-specific menopausal status is shown in patients aged from 40 to 60 years. **d** The subtypes of patients without metastasis (M0) are classified based on hormone receptor (HR) and human epidermal growth factor receptor 2 (HER2) status. HR-pos, hormone receptor-positive; estrogen receptor (ER)-positive or progesterone receptor (PgR)-positive. HR-neg, HR-negative; ER-negative and PgR-negative. HER2-pos, HER2-positive; HER2 immunohistochemistry (IHC) 3 + or IHC 2 + /in situ hybridization (ISH)-positive. HER2-neg, HER2-negative; HER2 IHC 0, 1 + or IHC 2 + /ISH-negative. HER2-zero; HER2 IHC 0, HER2-low; HER2 IHC 1 + or IHC 2 + /ISH-negative

Of the 102,453 breast cancer patients, 101,793 (99.4%) were female. A breakdown by stage of how the 101,793 female breast cancer cases were detected is shown in Fig. 1a. In stage 0, 51.3% of patients were detected by screening and 27.8% by self-detection. In stage I, 44.0% were detected by screening and 37.3% by self-detection, while in stage IV, 6.8% were detected by screening and 72.3% by self-detection. The demographics and characteristics of the women with breast cancer are shown in Table 1. Regarding the geographic distribution of these patients in Japan, 35.5% were from the Kanto region, 17.0% from the Chubu region, and 16.8% from the Kinki region (see Supplementary Table 1 for regional segmentation and data for each prefecture). The median age at cancer diagnosis was 62 years (interquartile range, 50–73 years). The distribution of the age at cancer diagnosis showed bimodal peaks at 45–49 years and 70–74 years (Fig. 1b). Bilateral breast cancer was found in 11,683 cases (11.5%), of which 7388 cases (7.3%) were synchronous and 4,345 cases (4.3%) were metachronous bilateral breast cancer (Table 1). Assessment of family history revealed that at least one first- or second-degree relative had a history of breast cancer in the NCD-BCR, accounting for 19.3% of cases. Among the female patients, 29.4% were premenopausal. Age-specific menopausal status from 40 to 60 years of age is shown in Fig. 1c. The proportion of patients with a body mass index (BMI) of 25% or greater was 27.3%, and 6.7% of patients had a BMI of 30% or greater. Of the 101,793 female patients, 15,437 (15.2%) and 42,936 (42.2%) were diagnosed with stage 0 and I disease, respectively, based on the TNM classifications using the Union for International Cancer Control staging system [21].

**Table 1:** Clinical characteristics of women with breast cancer in 2022

Characteristic	Total (N = 101,793)	
Geographical distribution, No. (%)		
Hokkaido	4532	(4.5)
Tohoku	6352	(6.2)
Kanto	36,103	(35.5)
Chubu	17,301	(17.0)
Kinki	17,052	(16.8)
Chugoku	5679	(5.6)
Shikoku	2906	(2.9)
Kyushu	11,861	(11.7)
Others	7	(0)
Age, years, median (interquartile range)	62	(50–73)
Unilateral or bilateral, No. (%)		
Unilateral	90,060	(88.5)
Bilateral, synchronous	7388	(7.3)
Bilateral, metachronous	4345	(4.3)
Family history, No. (%)		
Absence	77,743	(76.4)
Presence	19,608	(19.3)
Unknown	4345	(4.4)
Menstruation, No. (%)		
Premenopausal	29,917	(29.4)
Postmenopausal	69,251	(68.0)
Unknown	2,625	(2.6)
Body mass index, No. (%)		
< 18.5	10,150	(10.0)
≥ 18.5, < 25.0	63,004	(61.9)
≥ 25.0, < 30.0	20,999	(20.6)
≥ 30.0, < 35.0	5419	(5.3)
≥ 35.0, < 40.0	1117	(1.1)
≥ 40.0	286	(0.3)
Unknown	818	(0.8)
Clinical T status, No. (%)		
Tis	15,535	(15.3)
T0	414	(0.4)
T1	46,006	(45.2)
T2	30,278	(29.7)
T3	3202	(3.1)
T4	4883	(4.8)
Unknown	1475	(1.4)
Clinical N status, No. (%)		
N0	83,268	(81.9)
N1	12,745	(12.5)
N2	2042	(2.0)
N3	2225	(2.2)
Unknown	1513	(1.5)
Clinical M status, No. (%)		
M0	97,625	(95.9)
M1	2211	(2.2)
Unknown	1957	(1.9)
Clinical stage, No. (%)		
0	15,437	(15.2)
I	42,936	(42.2)
IIA	23,940	(23.5)
IIB	7933	(7.8)
IIIA	2326	(2.3)
IIIB	3225	(3.2)
IIIC	1612	(1.6)
IV	2211	(2.2)
Unknown	2173	(2.1)

TNM classifications were identified using the UICC staging system [21]

### Pathology

Pathological evaluation of surgical specimens from 97,154 patients, regardless of preoperative therapy, revealed that 12,028 (12.4%) patients had no invasive carcinoma (pTis) and the most common range of invasive tumor size was from 1.1 to 2.0 cm (Table 2). Axillary lymph node metastasis was found in 20.5% of patients. Estrogen receptor (ER), progesterone receptor (PgR), and human epidermal growth factor receptor 2 (HER2) were positive in 78.7%, 69.4%, and 12.8% of patients, respectively. Nuclear grade 3 was found in 14.8% of patients. The subtype classification based on being hormone receptor (ER and PgR) positive/negative and HER2 expression positive/negative in patients without metastasis (M0 disease) is shown in Fig. 1d. Furthermore, Fig. 1e shows the subtype classification based on being hormone receptor positive/negative and HER2 expression zero/low/positive in breast cancer patients with metastasis (M1 disease).

**Table 2:** Pathological findings of breast cancer patients without distant metastasis

Finding	Total (N = 97,154)	
Size of invasive carcinoma, No. (%)		
0 cm	12,028	(12.4)
> 0 cm, ≤ 0.5 cm	7614	(7.8)
> 0.5 cm, ≤ 1.0 cm	13,857	(14.3)
> 1.0 cm, ≤ 2.0 cm	28,383	(29.2)
> 2.0 cm, ≤ 3.0 cm	14,976	(15.4)
> 3.0 cm, ≤ 4.0 cm	5581	(5.7)
> 4.0 cm, ≤ 5.0 cm	5581	(2.7)
> 5.0 cm	3861	(4.0)
Unknown	8236	(8.5)
Number of lymph node metastasis, No. (%)		
0	68,922	(70.9)
1–3	14,712	(15.1)
4–9	3513	(3.6)
≥ 10	1650	(1.7)
Unknown for number of lymph node metastasis	22	(0.0)
No axillary surgery	6264	(6.4)
Unknown for axillary surgery	2071	(2.1)
Estrogen receptor, No. (%)		
Negative	15,326	(15.8)
1–9%	2850	(2.9)
≥ 10%	73,605	(75.8)
NA	3236	(3.3)
Unknown	2137	(2.2)
Progesterone receptor, No. (%)		
Negative	24,039	(24.7)
1–9%	7001	(7.2)
≥ 10%	60,478	(62.2)
NA	3457	(3.6)
Unknown	2179	(2.2)
HER2, No. (%)		
Negative	71,794	(73.9)
Positive	12,401	(12.8)
NA	10,058	(10.4)
Unknown	2901	(3.0)
Nuclear grade, No. (%)		
1	36,963	(38.0)
2	24,722	(25.4)
3	14,421	(14.8)
NA	12,205	(12.6)
Unknown	8843	(9.1)

*HER2* human epidermal growth factor receptor 2

### Treatment

**Fig. 2:**
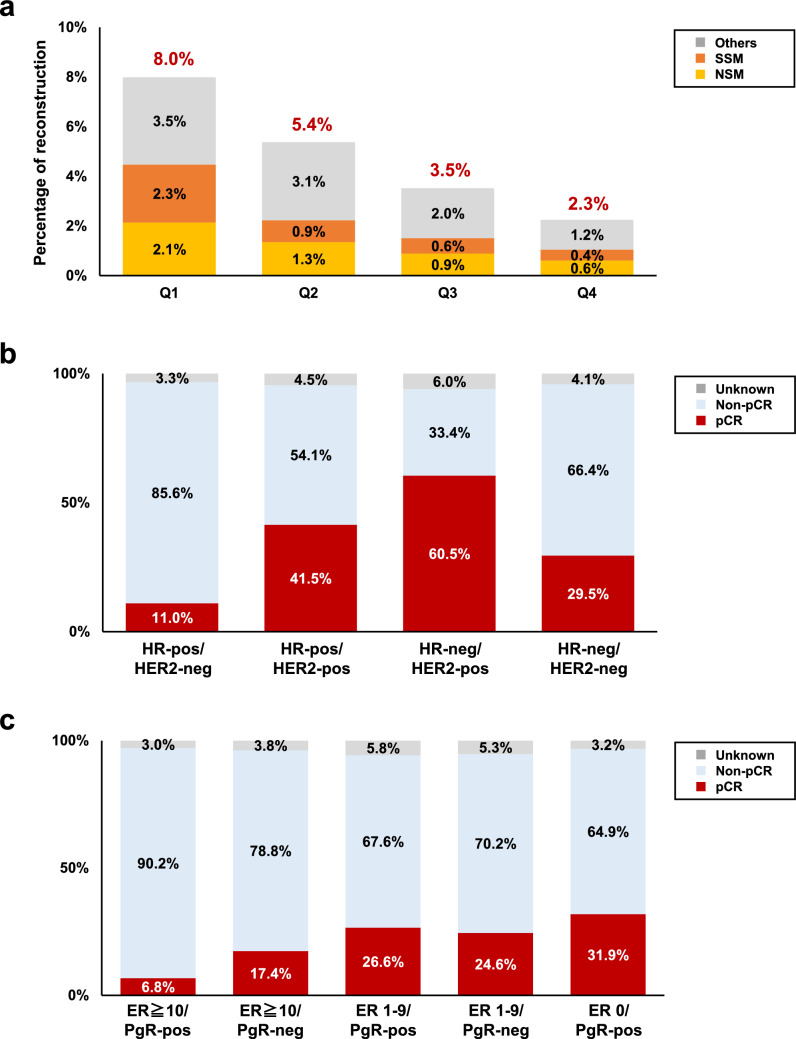
Clinical characteristics and the treatment of female patients with breast cancer. **a** The percentage of reconstruction among all breast surgeries by region, including the percentage of nipple-sparing surgery (NSM) or skin-sparing surgery (SSM) performed at the same time. Q1 represents the group of prefectures in the top quartile of recostruction implementation frequency, Q2 is the next quartile, followed by Q3 quartile, and Q4 is the group of prefectures in the bottom quartile. The numbers in red indicate the percentage of total reconstructions in each group, while the numbers in black indicate the percentages of NSM, SSM, and other surgeries performed for reconstruction. **b** Pathological complete response (pCR) rates in patients treated with preoperative chemotherapy by subtype are shown. **c** Details of pCR rates in patients with hormone receptor (HR)-positive, human epidermal growth factor receptor 2 (HER2)-negative who received preoperative chemotherapy are shown. HR-pos, hormone receptor-positive; estrogen receptor (ER)-positive or progesterone receptor (PgR)-positive. HR-neg, HR-negative; ER-negative and PgR-negative. HER2-pos, HER2-positive; HER2 immunohistochemistry (IHC) 3 + or IHC 2 + /in situ hybridization-positive. HER2-neg, HER2-negative; HER2 IHC 0, 1 + or IHC 2 + /in situ hybridization-negative. PgR-pos, PgR-positive. PgR-neg, PgR-negative

Of the 97,154 patients without distant metastasis, 40,521 (41.7%) underwent breast-conserving surgery (Table 3). Mastectomy was performed in 50,040 (51.5%) patients, including nipple-sparing mastectomy (NSM) and skin-sparing mastectomy (SSM) in 2171 (2.2%) and 1844 (1.9%) patients, respectively. A total of 66,894 (68.9%) patients were treated with sentinel lymph node biopsy (SLNB) and 7155 (7.4%) patients were treated with SLNB followed by axillary node dissection (Table 3). Among the 97,154 patients without distant metastasis, reconstruction was performed in 5,780 cases (5.9%; Table 3), and among the 54,055 cases with mastectomy (including NSM and SSM), reconstruction was performed in 5477 cases (10.1%; Table 4). When prefectures were ranked in order of the percentage of reconstruction performed, with the top 25% being the Q1 quartile, the next being the Q2 and Q3 quartiles, and the bottom 25% being the Q4 quartile, the percentage of reconstruction in the Q1 quartile was 8.0%, of which 2.1% was for NSM and 2.3% for SSM. In contrast, the percentage of reconstruction in the Q4 quartile was 2.3%, with 0.6% for NSM and 0.4% for SSM (Fig. 2a).

**Table 3:** Surgical procedure for breast cancer patients without distant metastasis

Procedure	Total (N = 97,154)	
Breast, No. (%)		
Breast-conserving surgery	40,521	(41.7)
Mastectomy	50,040	(51.5)
Nipple-sparing mastectomy	2171	(2.2)
Skin-sparing mastectomy	1844	(1.9)
Others	499	(0.5)
None	229	(0.2)
Unknown	6	(0.0)
Axilla, No. (%)		
Sentinel lymph node biopsy	66,894	(68.9)
Sentinel lymph node biopsy to axillary node dissection	7155	(7.4)
Axillary node dissection	13,766	(14.2)
Sampling	1004	(1.0)
Others	62	(0.1)
None	6264	(6.4)
Unknown	2009	(2.1)
Breast reconstruction, No. (%)		
None	89,109	(91.7)
Tissue expander	3695	(3.8)
Implant	276	(0.3)
Autologous	1503	(1.5)
Others	306	(0.3)
Unknown	5	(0.0)

**Table 4:** The relationship between the surgical procedure for the breast and the reconstructive procedure

Reconstructive procedure, No. (%)	Surgical procedure for the breast												Total (1 + 2 + 3 + 4 + 5)	
	1. Mastectomy		2. NSM		3. SSM		Subtotal (1 + 2 + 3)		4. Halsted		5. BCS			
None	47,608	(95.1)	639	(29.4)	329	(17.8)	48,576	(89.9)	308	(96.9)	40,225	(99.3)	89,109	(99.3)
Tissue expander	1819	(3.6)	901	(41.5)	916	(49.7)	3636	(6.7)	2	(0.6)	57	(0.1)	3695	(0.1)
Implant	33	(0.1)	176	(8.1)	66	(3.6)	275	(0.5)	0	(0)	1	(0)	276	(0)
Autologous (RAM flap)	221	(0.4)	180	(8.3)	217	(11.8)	618	(1.1)	0	(0)	16	(0)	634	(0)
Autologous (LDM flap)	281	(0.6)	217	(10.0)	246	(13.3)	744	(1.4)	8	(2.5)	117	(0.3)	869	(0.3)
Others	76	(0.2)	58	(2.7)	70	(3.8)	204	(0.4)	0	(0)	102	(0.3)	306	(0.3)
Unknown	2	(0)	0	(0.0)	0	(0.0)	2	(0.0)	0	(0)	3	(0)	5	(0)
Total	50,040	(100)	2171	(100)	1844	(100)	54,055	(100)	318	(100)	40,521	(100)	94,894	(100)

*NSM* nipple-sparing mastectomy, *SSM* skin-sparing mastectomy, *BCS* breast-conserving surgery, *RAM* rectus abdominis musculocutaneous, *LDM* latissimus dorsi musculocutaneous

Postoperative radiation therapy (RT) in patients without distant metastases is summarized in Table 5. In the group of patients treated with breast-conserving surgery (*n* = 40,521), 29,500 (72.8%) received whole-breast irradiation; RT to the supraclavicular fossa, internal mammary nodal region, or axilla was delivered to 1515 (3.7%), 236 (0.6%), and 952 (2.3%) patients, respectively. Among the patients who underwent mastectomy (*n* = 54,476), 6226 (11.4%) received RT to the chest wall; 5762 (10.6%), 1196 (2.2%), and 1072 (2.0%) patients received RT to the supraclavicular fossa, internal mammary nodal region, or axilla, respectively.

**Table 5:** Radiotherapy for breast cancer patients without distant metastasis

Procedure	No. of patients (%^a^)	
Breast-conserving surgery	Total (N = 40,521)	
Breast-conserving surgery with the following irradiation sites		
Whole breast	29,500	(72.8)
Partial breast	813	(2.0)
Boost to tumor bed	7129	(17.6)
Supraclavicular fossa	1515	(3.7)
Internal mammary nodal region	236	(0.6)
Axilla	952	(2.3)
None	8457	(20.9)
Mastectomy	Total (N = 54,476)	
Mastectomy with the following irradiation sites		
Chest wall	6226	(11.4)
Supraclavicular fossa	5762	(10.6)
Internal mammary nodal region	1196	(2.2)
Axilla	1072	(2.0)
None	45,235	(83.0)

^a^ percentage for total number of breast-conserving surgery or mastectomy

The types of systemic treatment in the pre- or post-operative therapies for patients without distant metastases are summarized in Table 6. Of the 13,950 patients who received preoperative chemotherapy with or without molecular targeted therapy, 4,308 (30.9%) achieved a pathological complete response (pCR). The highest pCR rate was 60.5% in patients with the HR-negative/HER2-positive subtype, while the lowest rate was 11.0% in patients with HR-positive/HER2-negative subtype (Fig. 2b). The pCR rates were 41.5% and 29.5% in the HR-positive/HER2-positive and triple-negative breast cancer (TNBC; HR-negative/HER2-negative subtype), respectively. The pCR rate in the HR-positive/HER2-negative population was further subdivided by ER (0, 1–9, 10 or more) and PgR (negative or positive), as shown in Fig. 2c.

**Table 6:** Pre- or post-operative therapies for breast cancer patients without metastasis

	Preoperative therapy		Postoperative therapy	
Therapy	Total (N = 18,429)		Total (N = 78,742)	
Endocrine therapy, No. (%)				
AIs	3195	(17.3)	37,805	(48.0)
SERMs	1177	(6.4)	17,416	(22.1)
AIs or SERMs + LHRHa	210	(1.1)	4525	(5.7)
Others	300	(1.6)	1107	(1.4)
Chemotherapy, No. (%)				
Anthracyclines	11,643	(63.2)	8172	(10.4)
Taxanes	12,305	(66.8)	8925	(11.3)
TC	303	(1.6)	3674	(4.7)
Capecitabine	35	(0.2)	1752	(2.2)
S-1	49	(0.3)	1373	(1.7)
Carboplatin	248	(1.3)	120	(0.2)
Others	558	(3.0)	1276	(1.6)
Molecular targeted therapy, No. (%)				
Trastuzumab	5293	(28.7)	7461	(9.5)
Pertuzumab	4770	(25.9)	4525	(5.7)
T-DM1	29	(0.2)	1439	(1.8)
Bevacizumab	156	(0.8)	41	(0.1)
Olaparib	6	(0.0)	279	(0.4)
Others	94	(0.5)	196	(0.2)

*AI* Aromatase inhibitors, *SERM* selective estrogen receptor modulator, *LHRHa* luteinizing hormone releasing hormone agonists, *TC* docetaxel and cyclophosphamide, *S-1* tegafur/gimeracil/oteracil, *T-DM1* trastuzumab emtansine

### Postscript

The detailed information on breast cancer in the NCD-BCR was volunteered by medical staff, including physicians in Japan. We appreciate their efforts to analyze the data, publish the paper, and have a great impact on the care of breast cancer patients. We hope that this annual report will help physicians and scientists understand trends in breast cancer characteristics and treatments in Japan.

## Supplementary Information

Below is the link to the electronic supplementary material.

Supplementary file 1 (DOCX 18 KB)

## Data Availability

The data that support the findings of this study are not openly available due to the nature of the clinical data used. The clinical data are derived from the registry, which is not an open database. The data were accessed by a designated statistician through an application process approved by academic societies. Therefore, we are unable to offer the original clinical data.
