# Supplementary material for: Breast cancer statistics for Japan in 2022: annual report of the national clinical database-breast cancer registry—clinical implications including chemosensitivity of breast cancer with low estrogen receptor expression
Source: Breast Cancer. 2025 Feb 6;32(2):217–26. doi: 10.1007/s12282-025-01671-0 (PMC11842403; doi:10.1007/s12282-025-01671-0)
Supplement: Supplementary file 1 — (DOCX 18 KB) [file 12282_2025_1671_MOESM1_ESM.docx]

| **Supplementary Table 1** Distribution of breast cancer patients according to prefecture | | | |
| --- | --- | --- | --- |
| Region | Prefecture | No. of patients | ％ |
| Hokkaido | 1. Hokkaido | 4,532 | 4.5 |
| Tohoku | 1. Aomori | 841 | 0.8 |
|  | 1. Iwate | 896 | 0.9 |
|  | 1. Miyagi | 1,869 | 1.8 |
|  | 1. Akita | 673 | 0.7 |
|  | 1. Yamagata | 844 | 0.8 |
|  | 1. Fukushima | 1,229 | 1.2 |
| Kanto | 1. Ibaraki | 2,306 | 2.3 |
|  | 1. Tochigi | 1,447 | 1.4 |
|  | 1. Gunma | 1,502 | 1.5 |
|  | 1. Saitama | 5,678 | 5.6 |
|  | 1. Chiba | 5,114 | 5.0 |
|  | 1. Tokyo | 12,396 | 12.2 |
|  | 1. Kanagawa | 7,660 | 7.5 |
| Chubu | 1. Niigata | 1,699 | 1.7 |
|  | 1. Toyama | 776 | 0.8 |
|  | 1. Ishikawa | 912 | 0.9 |
|  | 1. Fukui | 569 | 0.6 |
|  | 1. Yamanashi | 662 | 0.7 |
|  | 1. Nagano | 1,573 | 1.5 |
|  | 1. Gifu | 1,405 | 1.4 |
|  | 1. Shizuoka | 2,821 | 2.8 |
|  | 1. Aichi | 5,494 | 5.4 |
|  | 1. Mie | 1,390 | 1.4 |
| Kinki | 1. Shiga | 943 | 0.9 |
|  | 1. Kyoto | 2,178 | 2.1 |
|  | 1. Osaka | 7,410 | 7.3 |
|  | 1. Hyogo | 4,549 | 4.5 |
|  | 1. Nara | 1,130 | 1.1 |
|  | 1. Wakayama | 842 | 0.8 |
| Chugoku | 1. Tottori | 429 | 0.4 |
|  | 1. Shimane | 536 | 0.5 |
|  | 1. Okayama | 1,503 | 1.5 |
|  | 1. Hiroshima | 2,275 | 2.2 |
|  | 1. Yamaguchi | 936 | 0.9 |
| Shikoku | 1. Tokushima | 580 | 0.6 |
|  | 1. Kagawa | 738 | 0.7 |
|  | 1. Ehime | 1,049 | 1.0 |
|  | 1. Kochi | 539 | 0.5 |
| Kyushu | 1. Fukuoka | 4,517 | 4.4 |
|  | 1. Saga | 640 | 0.6 |
|  | 1. Nagasaki | 1,107 | 1.1 |
|  | 1. Kumamoto | 1,361 | 1.3 |
|  | 1. Oita | 932 | 0.9 |
|  | 1. Miyazaki | 879 | 0.9 |
|  | 1. Kagoshima | 1,304 | 1.3 |
|  | 1. Okinawa | 1,121 | 1.1 |
|  | 1. Others | 7 | 0.0 |
| Total |  | 101,793 | 100.0 |
